# Survival rates and bone loss after immediate loading of implants in fresh extraction sockets (single gaps). A clinical prospective study with 4 year follow-up

**DOI:** 10.4317/medoral.21651

**Published:** 2018-02-25

**Authors:** Eugenio Velasco-Ortega, Eduardo Wojtovicz, Antonio España-Lopez, Alvaro Jimenez-Guerra, Loreto Monsalve-Guil, Ivan Ortiz-Garcia, Maria-Angeles Serrera-Figallo

**Affiliations:** 1 Professor of Comprehensive Dentistry for Adults and Gerodontology. Faculty of Dentistry. University of Seville; 2 Clinical Professor of Comprehensive Dentistry for Adults and Gerodontology. Faculty of Dentistry. University of Seville; 3 Associate Professor of Comprehensive Dentistry for Adults and Gerodontology. Faculty of Dentistry. University of Seville; 4 Associate Professor of Comprehensive Dentistry for Special Patients. Faculty of Dentistry. University of Seville

## Abstract

**Background:**

The aim of this prospective study was to report the outcome of treatment with implants inserted after tooth extraction and immediately loaded.

**Material and Methods:**

Fifty-six patients with single tooth loss were treated with 116 IPX Galimplant® implants with internal connections and a sandblasted, acid-etched surface. All implants were placed after tooth extraction using a flapless approach without bone regeneration, and they were then immediately loaded with cemented acrylic prostheses. After a period of three months, definitive cemented ceramic prostheses were placed. Patients were examined throughout a total of 4 years of follow-up. Marginal bone loss and survival rates were evaluated using digital periapical radiographs, taking into account clinical variables such as age, gender, smoking, history of periodontitis, etiology of extraction, placement site, diameter, and implant length. The Mann-Whitney U and Kruskal-Wallis non-parametric tests were used to compare differences between subgroups created based on the different clinical variables identified.

**Results:**

Clinical results indicate an implant survival and success rate of 97.4%. Three implants were lost. Of the 116 immediate acrylic single crowns initially placed, 113 were replaced with definitive ceramic crowns after 3 months. A total of 77.8% of implants were inserted in the maxilla, while 22.2% were inserted in the mandible. No further complications were reported after the follow-up period (4 years). The mean marginal bone loss was 0.67 mm ± 0.40 mm. No differences were found among the subgroups of study patients.

**Conclusions:**

This study indicates that dental implants that are inserted after tooth extraction and immediately loaded may constitute a successful and predictable alternative implant treatment.

** Key words:**Dental implants, post-extraction implants, fresh sockets, immediate loading, immediate prostheses, implant dentistry.

## Introduction

Implant placement immediately following tooth extraction is a frequent clinical procedure and is considered as predictable as placing implants into healed sites (1-4). Immediate implant placement has its clinical advantages. Placing an implant in a fresh extraction socket may counteract the reabsorption of hard tissue and resulting reduction of the edentulous ridge. Treatment time is also reduced, and fewer surgical procedures are necessary when combining extraction, implant insertion, and bone grafting (if needed) into one appointment (5-8).

Implant placement in teeth sockets with periapical lesions may be contraindicated due to the potential for implant contamination during the initial healing period as a result of lingering infection. However, the high survival rates and normal crestal bone changes reported in several studies suggest that implants may be successfully osseointegrated when placed immediately after extraction in sockets with periapical infections, provided that appropriate preoperative procedures are taken to decontaminate the surgical sites (9-11).

In recent years, the outcomes of various implant treatments have been assessed, including implant success rates and the long-term stability of peri-implant tissues, measured using clinical and radiologic parameters (12,13). Botticelli *et al.* (12) reported the results of a prospective 5-year clinical study that demonstrated that implants installed in fresh extraction sockets and loaded after 5-7 months had a high success rate. During the follow-up observation period, no implants were lost, and the mean marginal bone level at the implant site was maintained or even improved.

Moreover, the immediate function protocol may also be an important measure for achieving improved aesthetic outcomes (7). Several studies have been conducted on the immediate loading of implants inserted in fresh sockets after extraction of compromised teeth (6-8,14). Crespi *et al.* (6) reported the 2-year follow-up findings of a comparative study assessing the immediate versus delayed loading of implants placed in maxillary sockets post-extraction. Forty patients who required one tooth extraction were treated with single implants; 20 immediately loaded implants were compared with 20 implants loaded after three months. The clinical outcomes reported a cumulative success rate of 100% for all implants (6). Barone *et al.* (7) compared the clinical and radiographic outcomes of immediate versus delayed restorations of single dental implants inserted in fresh extraction sockets after 2 years of function. Thirty patients were treated — 15 patients received immediate prosthetic restorations, and the remaining 15 were rehabilitated with a delayed restoration, which was carried out four months after extraction. After four years, no implants had failed. The two groups presented similar rates of successful tissue integration over the study period (7).

Several factors may affect the clinical outcomes of implants placed immediately in fresh extraction sockets (1,2) The implant’s design and surface both influence implant survival rates (8,15). Implant sites with a history of periodontal disease may pose an increased risk of implant failure (16). The differences in survival rates between implants placed in anterior and posterior sockets are controversial and may be attributable to variations in surgical protocol (17).

The aim of the present study is to evaluate the clinical outcomes of immediately loaded implants placed in fresh extraction sockets and to evaluate the relationship between crestal bone loss and age, gender, smoking, history of periodontitis, etiology of extraction, placement site, diameter, and implant length.

## Material and Methods

This prospective study included patients who had sought treatment at the clinic belonging to the Master’s in Oral Implantology of the School of Dentistry in Seville, Spain, and who required tooth extraction and replacement with dental prostheses between January 2011 and December 2015.

The study was conducted in accordance with the principles outlined in the Declaration of Helsinki on clinical research involving humans. The University of Seville’s ethics committee approved the study, and all patients provided informed written consent for immediate implant placement.

The study population consisted of 56 patients (treated consecutively), 28 females and 28 males, ranging in age from 33 to 63 years old (mean age of 48.7).

The following inclusion criteria were adopted: healthy patients with good oral hygiene, without chronic systemic diseases, and with only a single gap after tooth loss. Exclusion criteria included the presence of chronic systemic disease, smoking ≥ 10 cigarettes/day, bruxism, uncontrolled diabetes or periodontal disease, coagulation disorders, and alcohol or drug abuse.

All placement sites showed ≤ 5 mm of bone beyond the root apex to ensure primary implant stability, atraumatic extraction of the tooth, the integrity of the vestibular plate, and an insertion torque of ≥ 35 N.cm.

Treatment planning included diagnostic casts for intermaxillary relations, periapical and panoramic radiographs, and clinical photographs. Patients were informed of all possible implant choices for tooth replacement and accepted the immediate implant-supported prostheses.

One hour prior to surgery, the patients received prophylactic antibiotic therapy (500 mg amoxicillin and 125 mg clavulanic acid 1 hour before surgery); they also continued the treatment after the procedure, taking 3 capsules daily for 7 days. After surgery, a chlorhexidine mouthwash was prescribed for twice-daily use for 30 days. Ibuprofen (600 mg, 4 times daily) was prescribed for 7 days. All patients were treated under local anesthesia using articaine with adrenaline.

A flapless approach was chosen for the procedure, and tooth extractions were performed with elevators to help minimize trauma. Great care was taken to maintain the integrity of the buccal bone wall. After extraction, the socket was carefully curetted; subsequently, the implant bed was prepared according to the following procedure. None of the sites used in which teeth had been extracted due to caries or endodontic reasons presented any diffuse periapical lesions. The implant site was prepared using standard drills, following the palatal bony walls and always placed ≥ 4 mm beyond the root apex. The coronal margin of the implant was located at the buccal level of the bone crest. IPX® screw implants (Galimplant®, Sarria, Spain) with a sandblasted, acid-etched surface and internal connections were used for all implant placements. No grafting materials or barriers membranes were used.

After the surgical procedure, all patients immediately received abutments and temporary prosthetic restorations. Acrylic resin-cemented crowns were used for single-tooth replacements. Immediate loading was performed when an insertion torque of ≥ 35 N.cm was reached. The temporary crowns were removed three months after implant placement. The three implants lost at the beginning of the study did not receive any definitive restorations. Impressions were made of silicone material using open individual trays. Definitive metaloceramic restorations were cemented onto the osseointegrated implants.

The criteria used to assess survival rates were implant stability and the absence of radiolucency around the implants, mucosal suppuration, and pain. Follow-up visits were scheduled at 3 months after implant placement and at 1, 2, 3, and 4 years post implantation. During these check-ups, the patients’ prostheses and implants were cleaned and examined both clinically and radiologically. Marginal bone loss was evaluated based on digital periapical radiographs taken at a perpendicular angle to the long axis of the implants, assessing the difference between the 1-year follow-up radiography and the 4-year follow-up radiography. The following patient information was recorded: age, gender, smoking habits (< 10 cigarettes/day), history of periodontitis, placement site, diameter, implant length, and etiology of extraction. The unit of analysis was the patient for the first four variables, and the implant for the latter four.

The available data from all examinations were included in analyses using the SPSS (SPSS 11.5.0, SPSS, Chicago, USA) software package. Descriptive statistics were used to report the general results of the study as mean ± standard deviation. The Mann-Whitney U and Kruskal-Wallis non-parametric tests were used to compare differences between the groups created based on different measured risk factors. The level of significance was set at 5%.

## Results

One hundred sixteen implants were placed immediately after tooth extraction. The reasons for tooth extraction included caries and endodontic treatment failure, periodontal disease, and tooth fracture. The implant sites are presented in  Table 1.

**Table 1:**
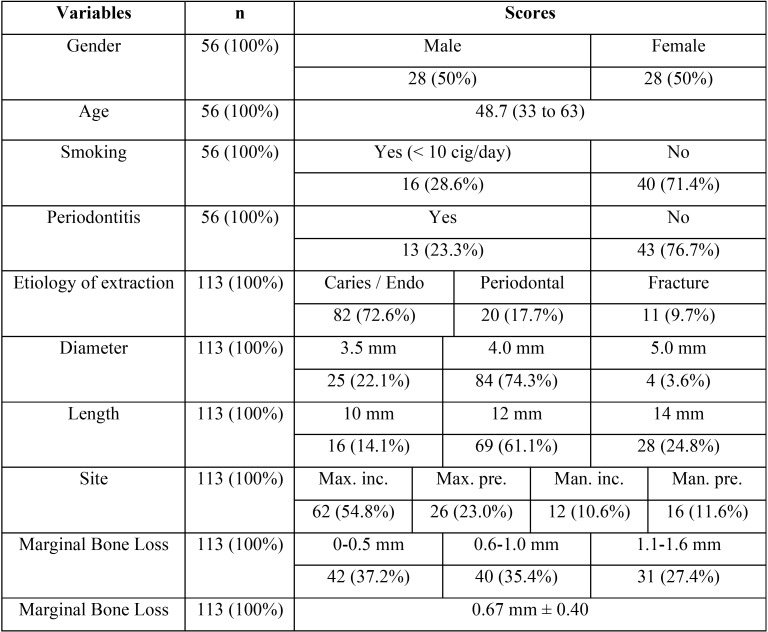
Characteristics of patients and implants included in the study.

In total, 3 implants failed during the initial healing period and were considered early failures. There were no signs of peri-implant infection during the follow-up period. The remaining 113 implants fulfilled the success criteria, and the cumulative success rate for all immediate implants was 97.4%.

The mean marginal bone loss was 0.67 mm (SD: 0.40 mm), ranging from 0 to 1.6 mm from the time of implant insertion to the 4-year follow-up evaluation ( Table 1). Of the implants placed in fresh extractions sockets, 14.2% showed no loss of marginal bone during the follow-up period. The proportion of implants with marginal bone loss of 0-0.5 mm was 37.2%.; 35.4% of the implants showed bone loss of 0.6-1.0 mm; and 27.4% of the implants showed marginal bone loss of 1.1-1.6 mm. Marginal bone losses were related to the clinical variables ( Table 2).

**Table 2:**
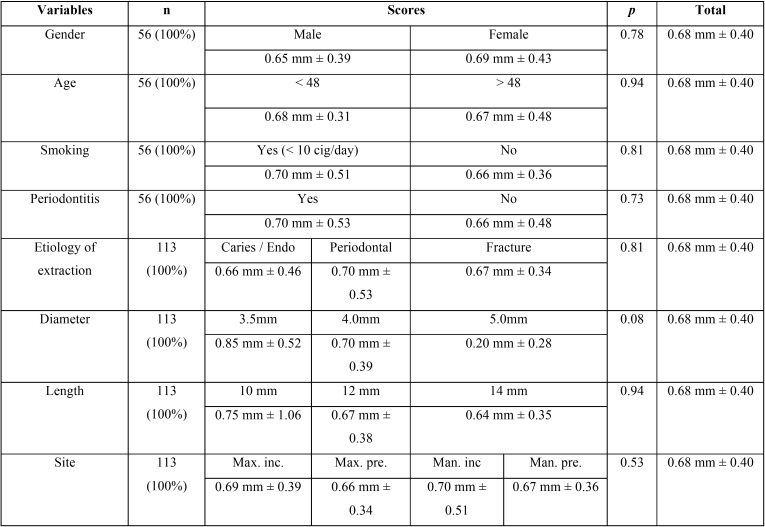
Marginal bone loss of implants in the study.

Temporary crowns were removed 3 months after implant insertion. Definitive prosthetic restorations consisted of ceramic single-cemented crowns. The prosthetic restorations were functional throughout the 4-year period, showing a cumulative success rate of 100%. There were no mechanical complications (e.g. fracture, loss of abutments and/or prosthetic screws).

## Discussion

Immediate insertion of implants in fresh extractions sockets has been documented and extensively discussed in the scientific literature. Advantages and disadvantages have been attributed to differences in protocol. The efficacy of these protocols in terms of enhancing the survival of implants inserted to restore extracted teeth and maintaining bone and soft tissue stability has been evaluated in recent systematic reviews (1,2,5,10,18-21).

In the present study, the bone healing process was successful for immediately loaded implants placed in fresh sockets for single rehabilitations. A total of 116 immediate implants were inserted, with a cumulative survival rate of 97.4%. All cases were treated with tooth extraction, flapless immediate placement, and immediate loading. The clinical findings from this 4-year follow-up study suggest that implants inserted immediately after tooth extraction and immediately loaded produce favorable outcomes and stable tissues conditions. Similar results were reported by several authors who evaluated the clinical success of implants immediately inserted in post-extraction sockets and immediately loaded with provisional prostheses (22-24). McAllister *et al.* (24) reported a cumulative survival rate of 98.3% after 2 years and significant improvements in patients’ self-esteem in a clinical study of 60 implants placed in 55 patients.24 Malchiodi *et al.* (23) showed an implant success rate of 100% in a 3-year prospective study of 64 maxillary single-tooth implants inserted immediately post-extraction and immediately loaded.

The use of membranes and/or grafting materials may provide an additional regenerative element for healing post-extraction implants placed in fresh sockets (3,14). However, this surgical technique does not appear necessary in all cases (8,13). In fact, generated circumferential defects healed clinically during the osseointegration period without membranes and/or grafting materials (25). The present study corroborated these results, as no additional materials were placed in the socket.

Marginal bone loss was considered an important clinical parameter in this study. Overall, crestal bone loss ranging from 0 to 1.6 mm was observed after 4 years. These clinical findings were in agreement with other studies showing that implants placed in fresh extraction sockets had an acceptable level of marginal bone stability (3,12,13). Among the studies reviewed, the highest percentage of marginal bone loss occurred over the first year of functioning, with bone levels becoming stable afterward. Moreover, half of the bone loss measured in the first year occurred within the first 3 months (24).

Smoking may be an important risk factor with adverse effects on implant survival and crestal bone loss (26). Several studies of post-extraction implants in fresh sockets included patients who smoked, but these did not report significant clinical findings (3,8,23). The present study found that smoking (up to 10 cigarettes a day) had no effect on the level of marginal bone loss. In fact, the mean crestal bone loss rates were 0.70 ± 0.51 mm (range: 0-1.2 mm) among patients who smoke and 0.66 ± 0.36 mm (range: 0-1.6 mm) among nonsmoking patients. Barbieri *et al.* (27) reported similar results in 20 patients treated with 120 maxillary post-extraction implants with immediate loading, showing that smoking had no effect on bone loss after a period of 18 months.

Marginal bone remodeling is a biological phenomenon that is influenced by several factors including macrogeometry and the microscopic surface of the implant (13,14,22,25,28). Several factors related to the length and diameter of implants may affect the clinical outcomes of post-extraction implants in fresh sockets. These features are important for primary stability (8,13,14,25). Wide implants may reduce the gap between the implant and the surrounding bone walls, (8,25) but they are more frequently placed in the molar regions, with a wider buccal cortical than the anterior region. Buccal cortical thickness has been shown to be more important than gap size when it comes to preventing bone loss (28).

The macroscopic design of implants is very important for primary stability and the load to bone (29-30). This aspect may be relevant to improving the initial stability of implants inserted in fresh sockets and immediately loaded. The most distinguishing feature of the implant design used in the present study is the thread configuration. The threads have grooves in them that allow for greater initial fixation and more rapid bone formation during the healing process. Moreover, the implants have a platform-switching design to maintain a good crestal bone level. This feature is useful for long-term bone and soft tissue stability, both of which are evident in this study (29) Kolinski *et al.* (14) reported stable bone and soft-tissue levels around similar macroscopic-design implants after 3 years of function. Sixty implants were inserted in 55 patients. All implants were placed in fresh extraction sockets and immediately loaded with provisional restorations. One implant failure was reported during the follow-up period. The study showed minimal peri-implant bone remodeling around the implant, which results in healthy papillae formation and soft tissue remodeling (14).

It is generally assumed that a roughened implant surface results in a stronger bone tissue response than a machined surface. Cells cultured on rougher surfaces tend to exhibit more differentiated osteoblasts than cells cultured on smoother surfaces (31). The implant surfaces in the present study were roughened using sandblasting and acid etching procedures. The treated surface was present up to the lateral border of the platform. In addition, the implants were inserted in sockets that were either level with or below the bone margin, which may explain the osseointegration that occurred up to the implant platform level. The clinical outcomes of this study indicate successful bone integration with the implants in extraction sockets, corroborating the reported results of similar clinical studies with treated implant surfaces (5,8,14). In a 5-year clinical follow-up study, Mura8 inserted 79 oxidized implants into fresh extraction sockets in 56 patients. All implants were immediately loaded with provisional restorations. No implants failed, resulting in an implant survival rate of 100%. The mean marginal bone loss from implant insertion to 5 years was 0.56 mm, indicating an overall stability of the peri implant bone (8).

After the surgical procedure, all patients immediately received abutments and temporary prosthetic restorations. Abutments were mounted directly on the internal cones of the implants and were not removed. The design of the implants allowed for a stable and passive connection between the implant and the abutment with optimal biomechanics. This aspect is important because the removal of implant abutments may deliver greater stress and interfere with the healing process, increasing marginal bone loss (32). At 3 months, the temporary crowns were replaced with definitive ceramic crowns. All restorations were single crowns placed paying careful attention to their emergence profile, occlusion, and prosthetic design. The outcomes of the present study demonstrate a 100% cumulative success rate of definitive prosthetic rehabilitation.

This 4-year follow-up clinical study showed that the immediate loading of implants placed in fresh extraction sockets demonstrates good treatment outcomes with regard to implant and prosthetics survival and marginal bone loss. Immediate loading of implants constitutes a clinically predictable treatment when strict selection criteria and clinical plans are applied.
